# Geographic variations and determinants of ever-tested for HIV among women aged 15–49 in Sierra Leone: a spatial and multi-level analysis

**DOI:** 10.1186/s12889-025-22079-7

**Published:** 2025-03-11

**Authors:** Lovel Fornah, Mulugeta Shegaze Shimbre, Augustus Osborne, Alieu Tommy, Agumas Fentahun Ayalew, Wei Ma

**Affiliations:** 1 School of Public Health, Cheeloo College of Medicine, Shandong University, Jinan, Shandong 250012 China; 2 Department of Public Health, Faculty of Basic Health Sciences, Ernest Bai Koroma University of Science and Technology, Makeni, Sierra Leone; 3 School of Public Health, College of Medicine and Health Sciences, Arba Minch University, Araba Minch, Ethiopia; 4 Institute for Development, IfD, Western Area, Freetown, Sierra Leone; 5 Department of Epidemiology, Southern Medical University, Guangzhou, China; 6 Department of Public Health, College of Medicine and Health Science, Injibara University, Injibara, Ethiopia

**Keywords:** HIV testing, Geographical variations, Spatial autocorrelation

## Abstract

**Background:**

HIV testing among women in sub-Saharan Africa varies widely, with Sierra Leone having lower rates than other countries. This study explores geographic variations and determinants of HIV testing among women aged 15–49 in Sierra Leone.

**Method:**

The study utilized data from the 2008, 2013, and 2019 Sierra Leone Demographic Health Surveys, comprising **39**,**606 women** aged 15–49. Spatial autocorrelation and Moran’s I were used to analyze the distribution of this outcome, while mixed-effect multi-level binary logistic regression assessed the factors associated with ever-tested for HIV. The findings were reported as adjusted odds ratios (aOR) with a 95% confidence interval (CI).

**Results:**

This study found that 21.47% of the study population comprised young women aged 15–19, and 53.62% had no formal education. Ever tested for HIV drastically increased from 13% in 2008 to 56% in 2019. HIV testing hotspots expanded from the Western urban and rural areas in 2008 to include districts like Port Loko, Kambia, and Bo by 2019, with a national testing pooled prevalence of 45.5% [44.2, 46.8]. The pooled regression analysis shows that women aged 20–34 had higher odds of testing than those aged 15–19, while those aged 40–49 had lower odds. Higher education, marriage/cohabitation, media exposure, parity, sexual activity, recent healthcare visits, condom use, STI history, larger households, female-headed households, and higher wealth indices were associated with higher odds of testing. Testing odds were higher during the 2013 and 2019 survey years compared to 2008. Conversely, Muslims, women with challenges accessing healthcare, and those in rural areas had lower odds of being tested.

**Conclusion:**

From 2008 to 2019, the rate of women aged 15–49 years ever tested for HIV showed a significant increase. The expansion of HIV testing hotspots highlights progress in geographic coverage, but disparities remain, particularly in rural areas. Younger women (aged 20–34) and those with higher education, wealth, or access to healthcare services were more likely to be tested, underscoring the influence of socioeconomic and structural factors on testing uptake. The lower odds of testing among older women and Muslims point to the need for targeted interventions addressing cultural barriers. Media exposure, parity, and sexual activity emphasize the role of reproductive health and awareness in promoting testing. Efforts to improve access to healthcare in rural areas and address logistical challenges, such as distance to health facilities, are critical for equitable HIV testing coverage. Strengthening community-based outreach and culturally sensitive programs could further close the gaps in testing uptake. The sustained increase in testing prevalence from 2008 to 2019 reflects progress but also highlights the need for continuous investment in HIV testing programs.

**Supplementary Information:**

The online version contains supplementary material available at 10.1186/s12889-025-22079-7.

## Introduction

Human Immunodeficiency Virus (HIV) remains a significant global health concern [1]. The most recent data from the 2023 UNAIDS Global HIV & AIDS Statistics Fact Sheet shows a further increase to 39 million people living with HIV globally, with an additional 1.3 million new cases [2]. Sub-Saharan Africa continues to bear the highest burden of HIV/AIDS, with women and children being disproportionately affected [3, 4]. In Sierra Leone, the HIV epidemic shows regional variations and continues to spread despite a low national HIV prevalence of 1.7% [5].

To achieve the objective of ending AIDS as a public health threat, the Joint United Nations Programme on HIV/AIDS set a target for 2025 to ensure that 95% of people living with HIV know their status [6]. Increasing the availability and uptake of HIV testing is crucial for reaching this goal [7]. HIV testing is vital for preventing HIV transmission, accessing antiretroviral treatment (ART), and receiving care and support [8, 9].

Conversely, Sierra Leone has fully implemented the testing guidelines provided by the World Health Organization to increase the uptake of HIV testing [10]. The nation has introduced a national HIV self-testing policy, expanded provider-initiated and targeted testing efforts, and implemented outreach and family testing alongside standard prenatal care services [11, 12].

However, in Sierra Leone, like in several other African countries, the rate of HIV testing uptake is low, particularly among women [13–18]. Approximately 40% of individuals with HIV are unaware of their status. Shockingly, 70% of young people diagnosed with HIV in Sierra Leone already have severe HIV illness when they make their first hospital visit [5]. Moreover, HIV distribution in Sierra Leone shows significant disparities between urban and rural areas [18, 19]. Urban residents are more likely to be HIV-positive and have been voluntarily tested for the virus [18]. Over 38% of women of reproductive age women have at least one HIV risk factor, with higher rates among younger women and those in urban and Northwestern regions [20].

In Sierra Leone, few studies [18, 21–24] have investigated HIV testing uptake, revealing low testing prevalence, with antenatal care as the primary method for women [18]. A 2017 study at an urban tertiary hospital found a high HIV prevalence of 24.3% among those tested and a low partner testing rate of just 0.4% despite a 52.9% positivity rate among partners who were tested [21].

Most of these studies have found a positive association between HIV testing and urban residence, previously married, those with higher education, condom use, early sexual debut, and unmarried individuals [5, 18, 20]. Nevertheless, none has focused on special populations like women and examined the regional variation of HIV testing in Sierra Leone. While it is true that women are particularly vulnerable to HIV/AIDS, it is essential to recognize that their vulnerability often arises from a range of factors, including social, economic, and cultural influences [16, 25, 26]. Therefore, addressing HIV/AIDS prevention requires a comprehensive approach that considers the behaviors and role of women. Examining the geographical variations and factors affecting HIV testing will help monitor the progress toward achieving ending AIDS, enable targeted resource allocation, and improve outreach for HIV testing services. This study aims to determine the geographical variations and factors influencing HIV testing among women aged 15–49 in Sierra Leone, utilizing data from the consecutive National Demographic and Health Surveys conducted in 2008, 2013, and 2019.

These DHS surveys, which form the basis of our analysis, do not collect data on HIV status for all respondents, nor does it provide a direct measure of the UNAIDS indicator (i.e., the proportion of people living with HIV who know their status). As such, “ever tested for HIV” was used as a proxy indicator to explore HIV testing behaviors in the general population. While not ideal, this indicator provides insights into testing uptake and access to testing services, which are critical to achieving the UNAIDS 95-95-95 targets. While lifetime testing may lose its value over time for individuals who remain at risk of HIV transmission, it is still a relevant measure for understanding progress in expanding access to HIV testing services, particularly in low-prevalence settings like Sierra Leone. Testing uptake is a necessary first step toward identifying people living with HIV and linking them to care.

Additionally, increasing testing rates can help reduce stigma, normalize HIV testing, and improve health-seeking behaviors in the general population. Sierra Leone’s National Strategic Plan on HIV/AIDS emphasizes the importance of increasing HIV testing coverage, especially in underserved populations and geographical areas with limited access to health services. By analyzing “ever tested for HIV,” we aim to identify disparities in testing access and highlight opportunities to improve testing strategies, particularly in regions or among disproportionately unreached populations.

## Methods

### Study design and sampling methods

Our analysis utilized data from women aged 15–49 who participated in the 2008, 2013, and 2019 Sierra Leone Demographic and Health Surveys (SLDHS). These surveys are household-based, nationally representative surveys employing a multistage cross-sectional design. The response rates for the individual women’s interviews were 95.1% in 2008, 96.0% in 2013, and 94.4% in 2019, ensuring high levels of participation and data quality.

More information can be found elsewhere for the SLDHS populations’ sampling procedures [27]. Briefly, the sample designs for each survey generally comprise two-stage probability samples drawn from an existing sample frame, typically the most recent census frame. In the 2008 and 2013 SLDHS, the pre-existing sample frame was from the population and housing censuses conducted in 2004, while the 2019 SLDHS used the 2015 Population and Housing Census as the pre-existing sample frame [28–30]. We combined the 2008, 2013, and 2019 SLDHS datasets into a single dataset to pool the surveys. Each survey was weighted using the sampling weights provided in the respective datasets to account for differences in sampling probabilities and ensure representativeness. The pooled dataset was then re-weighted to reflect the combined population structure across the three survey years. The final analyses involved a pooled weighted sample size of 39,606 women aged 15–49. This study conformed to the Strengthening Reporting of Observational Studies in Epidemiology (STROBE) guidelines [31].

### Data extraction and manipulation

After thoroughly reviewing the relevant documentation for each survey year, the SLDHS datasets for 2008, 2013, and 2019 were obtained in STATA format from the official Measure DHS program website (http://www.dhsprogram.com). For data cleaning, recoding, merging, and integrating the HIV-related datasets with the Global Positioning System (GPS) coordinates from the SLDHS, STATA version 18.0 was employed. This process adhered to the standard guidelines and procedures established by the Measure DHS program for effective data management and analysis.

### Measure variables

#### Outcome variable

The current study used ever-tested for HIV as the outcome variable. In the DHS dataset, participants were asked the question, “Have you ever been tested for HIV/AIDS?” and were given the option to answer “Yes” or “No”. We then coded the responses as “No = 0” and “Yes = 1”.

#### Explanatory variables

The explanatory variables for this analysis were selected based on their availability in the dataset and an in-depth review of previous studies [13–16, 18, 32, 33]. The variables considered in this study were categorized into individual and community variables.

However, we classified variables such as potential levers for increasing testing (e.g., media exposure, access to health facilities), Indicators of populations disproportionately unreached (e.g., region, age, education, marital status), and structural barriers: e.g., distance to health facilities, healthcare infrastructure.

The individual level variables were respondent’s age, educational attainment, employment status, marital status, religion, *household* wealth index, awareness of STIs, number of sexual partners, age at first sex, and number of children. Table 1 shows the coding scheme for these individual-level variables.

**Table 1:** Prevalence of ever-tested for HIV among women in Sierra Leone, 2008, 2013, and 2019

Variable	Survey Years			
	SLDHS 2008	SLDSH 2013	SLDHS 2019	Pooled total
**Total**,** n (%)**	7,374 (19%)	16,658 (42%)	15,574 (39%)	39,606 (100%)
**Ever-Tested HIV**				
No	6,437 (87.3%)	8,338 (50.1%)	6,810 (43.7%)	21,585 (54.5%)
Yes	937 (12.7%)	8,320 (49.9%)	8,764 (56.3%)	18,021 (45.5%)

^**†**^ SLDHS, Sierra Leone demographic and health survey; HIV, Human immunodeficiency virus

The community-level variables were *household* wealth index, residence, region, household size, and community media exposure. We used the *household wealth index* provided in the SLDHS datasets for 2008, 2013, and 2019. Following standard DHS methodology, the DHS program constructed the wealth index using principal component analysis (PCA) based on household asset ownership, housing characteristics, and access to services. We did not recompute the wealth index across surveys, as the index was pre-calculated within each survey. We classified the respondent’s residences into rural and urban areas. Community media exposure was defined as respondents’ prior history of media access. We created this variable from four questions that suggest media access: reading magazines or newspapers, listening to radio, watching television, or using the internet. We coded the response as “0 = no” if an individual had no access to any media and as “1 = yes” if they had access to at least one of these media [34].

### Statistical analysis

We conducted the statistical analyses using Stata version 18.0. We weighted the data for the cluster (enumeration areas), primary sampling unit (individual enumeration area), and strata (urban and rural areas within the administrative districts) prior to the statistical analysis. We did this to guarantee that the survey results accurately reflected the population and to guide the STATA program in adjusting for the intricate sampling design. First, we conducted a descriptive analysis to observe the weighted frequency and percentage. Subsequently, a bivariate analysis determined the distribution of HIV testing across the explanatory variables. We presented the results in a table, which displayed the proportion of individuals who had ever tested for HIV, their respective confidence intervals (CIs), and a p-value to signify their significance level.

### Spatial analysis

We conducted spatial analyses using ArcGIS Version 4.3.1 to identify the HIV testing rate and evaluate its statistical significance. We downloaded Shapefiles for each survey year from the DHS Spatial Data Repository [35]. Supplementary file-1 provides details of the spatial analysis.

### Multi-level binary logistic regression

In this study, we conducted a mixed-effect multi-level binary logistic regression analysis to examine the factors associated with being tested for HIV. The mixed-effect multi-level binary logistic regression analysis details are provided in Supplementary file-1.

### Ethical considerations

This study did not require ethical approval as the dataset was publicly available. Before using the dataset, we obtained permission from MEASURE DHS to access the SLHDS. For additional information regarding the DHS’s data usage practices and associated ethical considerations, please visit https://dhsprogram.com/Methodology/Protecting-the-Privacy-of-DHS-Survey-Respondents.cfm.

## Results

### Background characteristics of the women in Sierra Leone using pooled datasets 2008,2013 and 2019

The largest age group was 15–19-year-old women (21.47%). A significant portion, 53.62%, had no education, and the majority, 66.01%, were married/cohabiting. Despite this educational attainment, economic opportunities seemed high, with 69.13% working. Additionally, access to media and healthcare was low, with 97.41% not exposed to media and 97.73% lacking health insurance. While most,60.46%, had two or more children, a concerning number, 59.35%, reported having sex before 18, and over half, 51.86%, had not visited a health facility in the past year. Close to a quarter,23.69%, belonged to the richest wealth category, and over half,60.23%, resided in rural areas (Supplementary file 2: Table s1).

### Prevalence of ever tested for HIV among women in Sierra Leone, 2008, 2013 and 2019

The overall prevalence of women who reported having ever-tested for HIV across the three survey years was 45.5%. Specifically, in 2008, 12.7% of women indicated they had ever been tested for HIV, while this figure rose to 49.9% in 2013 and further increased to 56.3% in 2019 (see Table 1).

### Spatial pattern of ever-tested for HIV

Our analysis revealed that the spatial distribution of ever-tested for HIV among women aged 15–49 was not random in any of the survey years. The spatial autocorrelation analysis reveals the existence of statistically significant clusters at a significant level of 0.01 in each survey (Supplementary File 3: Table S1).

**Fig. 1:**
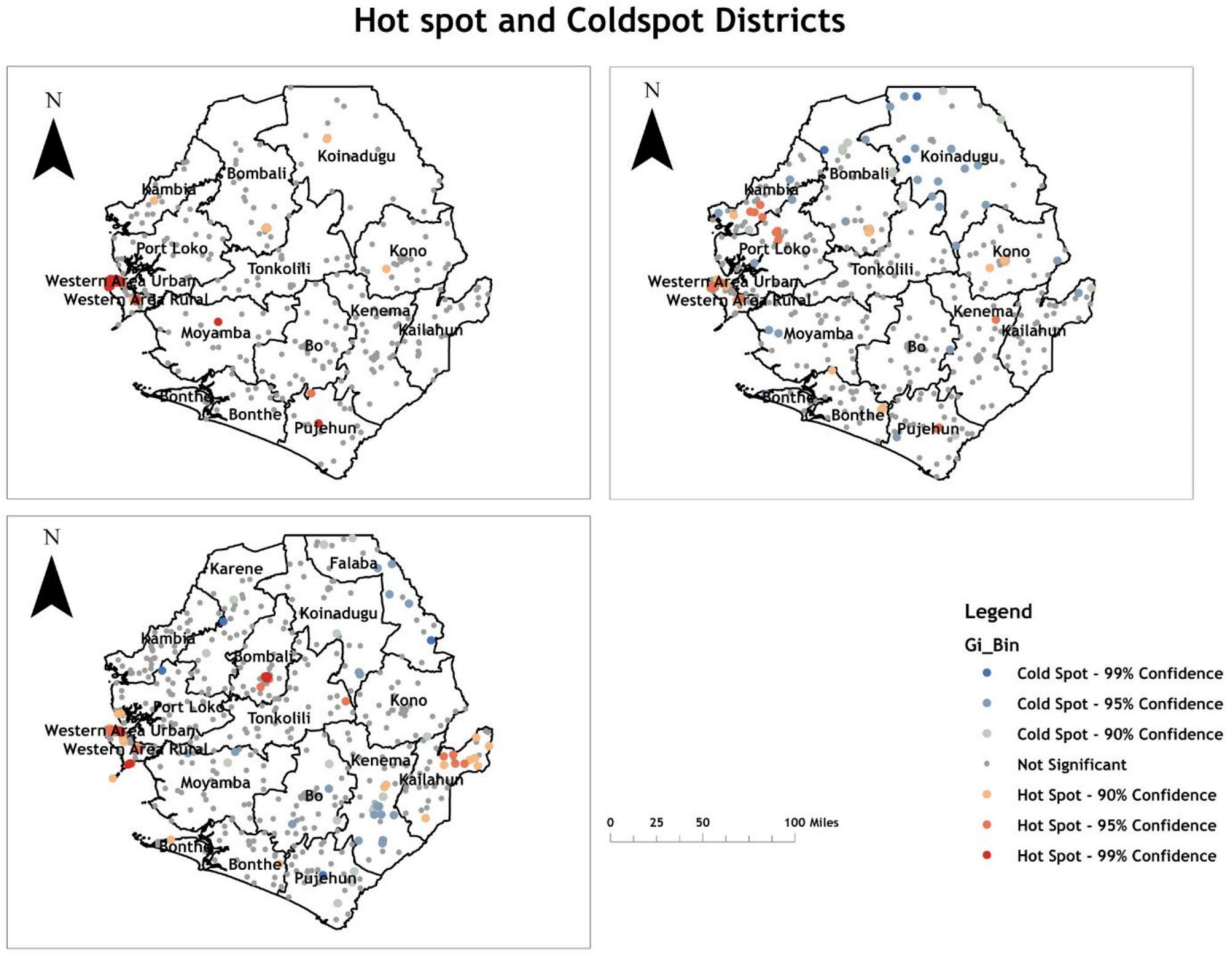
Hot spot and cold spot of women aged 15-49 for being ever-tested for HIV in Sierra Leone in 2008 (upper left panel), 2013 (upper right panel), and 2019 (lower left panel). HIV: Human immunodeficiency virus; SLDHS: Sierra Leone Demographic and Health Survey. Source: Spatial Data Repository, The Demographic and Health Surveys Program. ICF International. Funded by the United States Agency for International Development (USAID). Available from https://spatialdata.dhsprogram.com. [Accessed 01 August 2024] [32]

In the 2008 SLDHS, the Western area, a few areas of the rural area, Moyamba, and Pujehun districts were found to be statistically significant hotspot districts for being ever-tested for HIV. Similarly, Kono, Bombali, Koinadugu, and Kambia districts are hotspots at a 90% Confidence level. Conversely, Bonthe, Kenema, Kailahun, Port Loko, Bo, and Tonkolili districts were significant cold spots (Fig. 1 upper left panel).

In the 2013 SLDHS, the western area urban, western area rural, Kambia, southern Bombali, Pujeahun, central Kono, Port Loko, Bonthe, and a few areas of Kenema districts were statistically significant hotspot districts for being ever-tested for HIV. In contrast, some parts of Bombali, Koinadugu, Kailahun, Moyamba, Tonkolili, and Bo districts were statistically significant cold spots for being ever-tested for HIV (Fig. 1 upper right panel).

In the 2019 SLDHS, the western area urban, the western area rural, part of Kailahun, some parts of Bombali, some parts of Kenema, and some parts of Tonkolili districts show statistically significant hotspots for being ever-tested for HIV. Karene, Falaba, Koinadugu, Bo, some parts of Tonkolili, some parts of Kenema, Kono, Moyamba, Bonthe, Pujehun, and Port Loko districts were statistically significant cold spots for being ever-tested for HIV (Fig. 1 lower left panel).

**Fig. 2:**
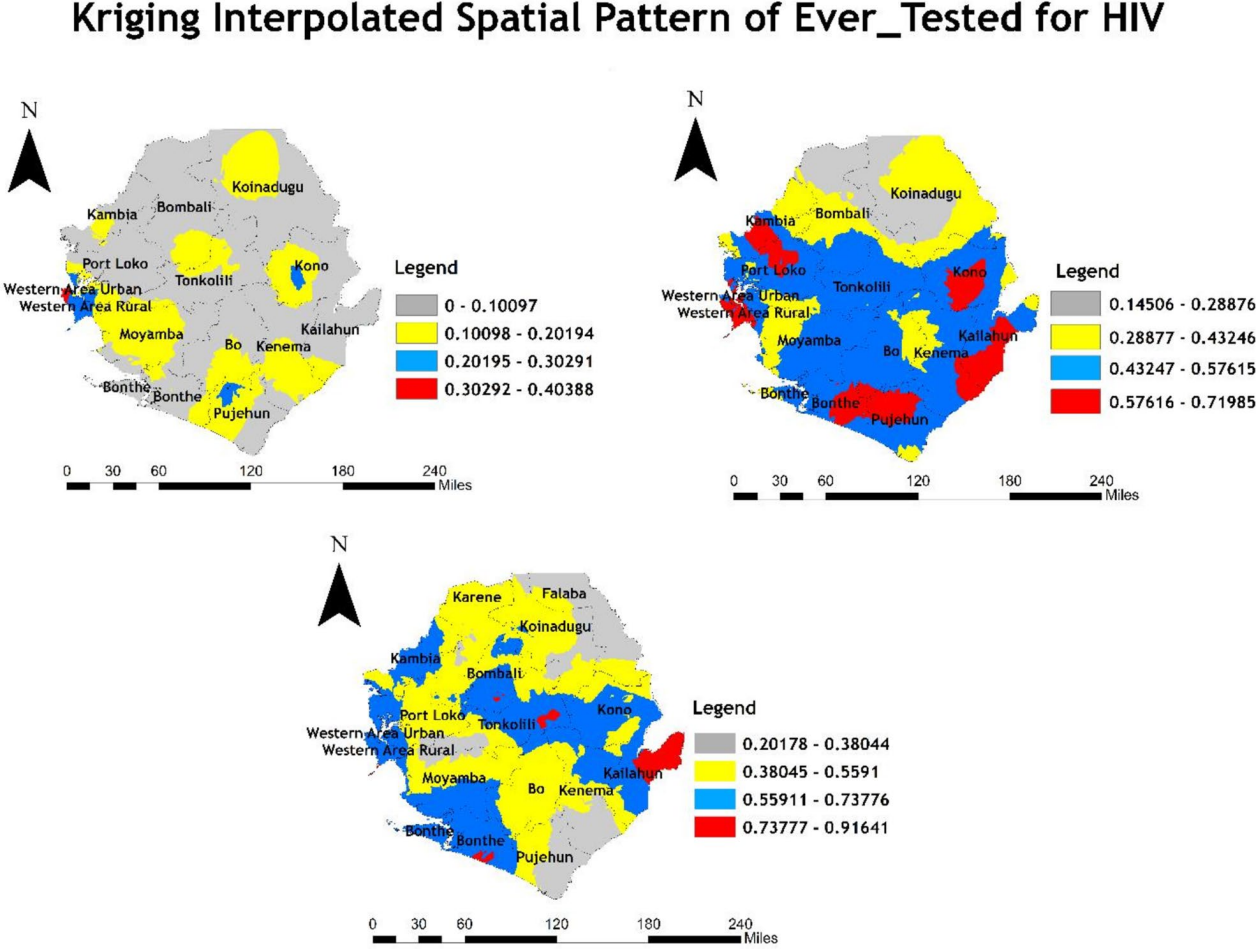
Kriging Interpolated spatial pattern of ever-tested for HIV among women aged 15-49 years in Sierra Leone in 2008 (upper left panel), 2013(upper right panel), and 2019 (lower left panel). HIV: Human Immubodeficiency virus SLDHS: Sierra Leone Demographic and Health Survey. Spatial Data Repository, The Demographic and Health Surveys Program. ICF International. Funded by the United States Agency for International Development (USAID). Available from: https://spatialdata.dhsprogram.com/ [Accessed 01 August 2024] [32]

The result of Empirical Bayesian Kriging (EBK) maps the estimated spatial pattern of ever-tested for HIV among women aged 15–49 years by interpolating the data to places where data were not collected. The districts with the highest estimated rates of being ever-tested for HIV in 2008 were the urban Western area, the rural area, eastern Kono, and central Pujehun. Conversely, the districts with the lowest rates of being ever-tested for HIV were Bombali, Bo, Bonthe, Kenema, Kailahun, Tonkolili, Koinadugu, Kambia, Port Loko, the north-west area of Kono, and Moyamba (Fig. 2 upper left panel).

In 2013, the Western area urban, the Western area rural, Port Loko, Kambia, Kono, Tonkolili, Moyamba, Bonthe, Bo, Kailahun, and Pujehun districts had higher rates of being ever-tested for HIV. Bombali, Koinadugu, Kenema, and a few areas of Moyamba districts exhibited lower rates of being ever-tested for HIV (Fig. 2 upper right panel).

In 2019, the Western area, the Western area rural, Western Port Loko, Kambia, Bonthe, Kailahun, Tonkolili, and a few areas of Bombali and Kono districts had greater rates of ever-testing for HIV. Karene, Falaba, Koinadugu, Bo, Kenema, Pujehun, and a few areas in Bombali and Port Loko districts had lower rates of ever-testing for HIV (Fig. 2 lower left panel).

### Bivariate results of the association between the explanatory variables and ever-tested HIV

We present the bivariable analysis of the pooled prevalence and distribution of ever-tested for HIV among women aged 15–49 years in Sierra Leone (Supplementary file 2: Table S2).

### Factors associated with ever-tested for HIV among women in Sierra Leone

Table 2 shows the factors associated with ever-tested for HIV among women in Sierra Leone. Women aged 20–24 [aOR = 1.47, 95% CI = 1.30, 1.67], 25–29 [aOR = 1.45, 95% CI = 1.27, 1.67], and 30–34 [aOR = 1.28, 95% CI = 1.10, 1.50] had higher odds of being ever tested for HIV than those aged 15–19 from model IV. Women with primary [aOR = 1.26, 95% CI = 1.14, 1.39], secondary [aOR = 1.78, 95% CI = 1.61, 1.98], and higher education [aOR = 3.73, 95% CI = 2.97, 4.71] had higher odds of being ever tested for HIV than those with no education. Women who were married/cohabiting [aOR = 1.18, 95% CI = 1.04, 1.32] had higher odds of being ever tested for HIV than those never in a union. Women who were exposed to the media [aOR = 1.51, 95% CI = 1.21, 1.90] had higher odds of being ever tested for HIV than those who did not. Women with one [aOR = 7.47, 95% CI = 6.39, 8.74] and two or more parity [aOR = 8.40, 95% CI = 7.12, 9.91] had higher odds of being ever-tested for HIV than those with no parity. Women who had sex below 18 [aOR = 3.82, 95% CI = 2.99, 4.89] and those who had sex 18+ [aOR = 4.18, 95% CI = 3.21, 5.45] had higher odds of being ever-tested for HIV than those who hadn’t had sex. Women who visited health facilities in the past 12 months [aOR = 2.08, 95% CI = 1.93, 2.25] had higher odds of being ever tested for HIV than those who did not. Women who used condoms [aOR = 1.41, 95% CI = 1.05, 1.89] had higher odds of being ever tested for HIV than those who did not. Women who had STI [aOR = 1.25, 95% CI = 1.09,1.44] had higher odds of being ever tested for HIV than those who didn’t. Women who were six or more in household size [aOR = 1.09, 95% CI = 1.01, 1.16] had higher odds of being ever tested for HIV than those in five and below. Women with households with female heads [aOR = 1.09, 95% CI = 1.01,1.17] had higher odds of being ever tested for HIV than those with males. Women in the middle [aOR = 1.13, 95% CI = 1.00, 1.28], richer [aOR = 1.25, 95% CI = 1.09, 1.43], and richest wealth index [aOR = 1.52, 95% CI = 1.27, 1.82] had higher odds of being ever tested for HIV than those in the poorest wealth index. Women in the 2013 [aOR = 10.77, 95% CI = 8.91, 13.01] and 2019 survey years [aOR = 15.56, 95% CI = 12.62, 19.19] had higher odds of being ever-tested for HIV than those in the 2008 survey year. Women aged 40–44 [aOR = 0.63, 95% CI = 0.53, 0.75] and 45–49[aOR = 0.36, 95% CI = 0.30, 0.43] had lower odds of being ever-tested for HIV than those aged 15–19. Women who were Muslims [aOR = 0.83, 95% CI = 0.75, 0.91] had lower odds of being ever tested for HIV than those who were Christians. Women with big problems with distance to health facilities [aOR = 0.87, 95% CI = 0.75, 1.00] had lower odds of being ever tested for HIV than those who didn’t. Women living in rural areas [aOR = 0.74, 95% CI = 0.63, 0.87] had lower odds of being ever tested for HIV than those living in urban areas.

**Table 2:** Factors associated with ever-tested for HIV among women aged 15–49 years in Sierra Leone, 2008,2013 and 2019 (*N* = 39,606)

Variables	Model I Empty model	Model II aOR [95% CI]	Model III aOR [95% CI]	Model IV aOR [95% CI]
**Fixed effect results**				
15–19		1.00		1.00
20–24		1.64***[1.45,1.85]		1.47*** [1.30,1.67]
25–29		1.62***[1.41,1.86]		1.45^***^ [1.27,1.67]
30–34		1.61***[1.37,1.89]		1.28***[1.10,1.50]
35–39		1.27***[1.08,1.50]		0.09 [0.79,1.09]
40–44		0.92 [0.77,1.10]		0.63***[0.53,0.75]
45–49		0.59***[0.49,0.71]		0.36***[0.30,0.43]
**Educational attainment**				
No education		1.00		1.00
Primary		1.38***[1.25,1.53]		1.26***[1.14,1.39]
Secondary		2.36***[2.12,2.62]		1.78***[1.61,1.98]
Higher		5.22***[4.17,6.53]		3.73***[2.97,4.71]
**Current working status**				
Not working		1.00		1.00
Working		0.94 [0.86,1.03]		0.95 [0.87,1.04]
**Marital status**				
Never in union		1.00		1.00
Married/cohabiting		0.96 [0.86,1.09]		1.18** [1.04,1.32]
Previously married		0.83*[0.71,0.98]		0.95 [0.80,1.13]
**Media exposure**				
No		1.00		1.00
Yes		1.15 [0.94,1.42]		1.51***[1.21,1.90]
**Covered by Health insurance**				
No		1.00		1.00
Yes		1.26 [0.97,1.62]		1.10 [0.82,1.48]
**Parity**				
Zero		1.00		1.00
One		6.06***[5.23,7.02]		7.47***[6.39,8.74]
Two or more		6.41***[5.46,7.52]		8.40***[7.12,9.91]
**Age at first sex**				
Not had sex		1.00		1.00
Below 18 years		3.64***[2.87,4.61]		3.82***[2.99,4.89]
18+		3.32***[2.58,4.28]		4.18***[3.21,5.45]
**Religion**				
Christians		1.00		1.00
Muslims		0.89*[0.80,0.99]		0.83*** [0.75,0.91]
Others		0.69 [0.43,1.10]		0.95 [0.63,1.44]
**Visited health facility last 12 months**				
No		1.00		1.00
Yes		2.28***[2.10,2.47]		2.08***[1.93,2.25]
**Multiple sexual partners**				
No		1.00		1.00
Yes		1.12 [0.97,1.30]		1.12 [0.96,1.31]
**Condom use**				
No		1.00		1.00
Yes		1.29 [0.98,1.69]		1.41* [1.05,1.89]
**Had STI**				
No		1.00		1.00
Yes		1.34***[1.16,1.54]		1.25**[1.09,1.44]
**Genital discharge**				
No		1.00		1.00
Yes		1.06 [0.95,1.18]		1.06 [0.96,1.29]
**Genital sore**				
No		1.00		1.00
Yes		0.87*[0.77,0.99]		0.94 [0.83,1.07]
**Distance to a health facility**				
No problem		1.00		1.00
Big problem		0.57***[0.50,0.64]		0.87*[0.75,1.00]
**Household size**				
Five and below		1.00		1.00
Six or more		1.01 [0.95,1.09]		1.09* [1.01,1.16]
**Sex of household head**				
Male		1.00		1.00
Female		1.02 [0.96,1.08]		1.09*[1.01,1.17]
***Household*** **wealth index**				
Poorest			1.00	1.00
Poorer			1.05 [0.95,1.15]	1.06 [0.96,1.17]
Middle			1.08 [0.96,1.21]	1.13* [1.00,1.28]
Richer			1.09 [0.95,1.25]	1.25** [1.09,1.43]
Richest			1.07 [0.91,1.27]	1.52***[1.27,1.82]
**Residence**				
Urban			1.00	1.00
Rural			0.90 [0.74,1.09]	0.74***[0.63,0.87]
**Region**				
Eastern			1.00	1.00
Northern			0.73**[0.59,0.91]	0.88[0.73,1.08]
Northwestern			0.97 [0.78,1.20]	1.15 [0.96,1.38]
Southern			1.09 [0.89,1.32]	1.21 [0.99,1.46]
Western			2.26***[1.81,2.82]	1.31[0.99,1.72]
**Survey years**				
2008				1.00
2013				10.77***[8.91,13.01]
2019				15.56***[12.62,19.19]
**Random effect model**				
PSU variance (95% CI)	0.36 [0.30, 0.44]	0.47 [0.39, 0.56]	0.30 [0.24, 0.37]	0.37 [0.30, 0.46]
ICC	0.10 [0.08, 0.11]	0.12 [0.10, 0.14]	0.08 [0.07, 0.10]	0.10 [0.08, 0.12]
Wald chi-square	Reference	2989.64***	185.12***	3311.88***
**Model fitness**				
Log-likelihood	-26284.469	-21739.499	-26005.027	-19419.28
AIC	52572.94	43,539	52034.05	38922.56
N	39,606	39,606	39,606	39,606
Number of clusters	578	578	578	578

aOR = adjusted odds ratios; CI = Confidence Interval; * p<; 0.05, ** p<; 0.01, *** p<; 0.001; 1.00 = Reference category; PSU = Primary Sampling Unit; ICC = Intra-Class Correlation; AIC = Akaike’s Information Criterion

## Discussion

This study aimed to examine the geographic variations and determinants of HIV testing among women aged 15–49 in Sierra Leone using data from the Sierra Leone Demographic and Health Surveys (SLDHS) of 2008, 2013, and 2019. By employing a spatial and multi-level analysis, we have uncovered significant insights into the factors influencing HIV testing and the spatial patterns of testing behaviors across the country.

Our findings reveal a significant increase in the percentage of women aged 15–49 in Sierra Leone who have ever been tested for HIV, increasing from 13% in 2008 to 56% in 2019. This trend is consistent with other studies demonstrating improved access to HIV testing globally [9, 36]. Several factors may contribute to this positive development, including the initiatives implemented by the government and non-governmental organizations to increase awareness about HIV testing and reduce the stigma associated with the disease. Additionally, improved access to healthcare services and increased availability of testing facilities may have also contributed to this positive trend in HIV testing rates among women in Sierra Leone.

Our findings show that ever-tested for HIV among women in Sierra Leone are not randomly distributed. Clusters of high and low testing rates were found in different regions and survey years. The Western urban area consistently emerged as a hotspot for ever-tested for HIV across all survey years, highlighting its role as a critical region for public health interventions. This finding aligns with previous research in sub-Saharan Africa, where urban areas often show higher testing rates due to better access to healthcare facilities and services [37].

In the 2008 SLDHS, hotspots for individuals ever-tested for HIV were found in the Western urban area, a few parts of the Western rural area, Moyamba, and Pujehun. Furthermore, a 90% confidence level identified Kono, Bombali, Koinadugu, and Kambia districts as hotspots and Bonthe, Kenema, Kailahun, Port Loko, Bo, Tonkolili, and Koinadugu districts as cold spots. By 2013, additional hotspots emerged in Kambia, southern Bombali, Pujehun, central Kono, and a few areas of Kenema districts, while coldspot persisted in Bombali, Koinadugu, Kailahun, Moyamba, Tonkolili, and Bo districts. In 2019, hotspots remained in the Western urban and rural areas, while cold spots expanded to new districts of Karene and Falaba. The consistent pattern of Western urban and rural areas being hotspot areas for ever-tested for HIV can be attributed to the better access to healthcare facilities, higher awareness campaigns, and robust HIV testing services in these areas [18]. However, districts such as Bombali, Koinadugu, and Tonkolili frequently appeared as cold spots, possibly reflecting barriers to accessing HIV testing services in these districts. These barriers may include distance to health facilities, lack of transportation, and lower health literacy, as noted in other studies in similar settings [38–40]. The Empirical Bayesian Kriging (EBK) results highlighted spatial patterns by estimating the ever-tested for HIV rates in areas where data were not collected. In 2008, the highest estimated testing rates were in the Western urban area, the western rural area, eastern Kono, and central Pujehun, while the lowest were in Bombali, Bo, Bonthe, Kenema, Kailahun, Tonkolili, Koinadugu, Kambia, Port Loko, the north-west area of Kono, and Moyamba. In 2013, the highest estimated testing rates were in the Western area urban, Western area rural, Port Loko, Kambia, Kono, Tonkolili, Moyamba, Bonthe, Bo, Kailahun, and Pujehun districts, while the lowest was in Bombali, Koinadugu, Kenema, and a few areas of Moyamba districts. By 2019, high testing rates persisted in the Western areas and other urban districts, while low rates remained in rural districts such as Karene, Falaba, and Koinadugu. These findings are consistent with previous studies that found that rural residents are less likely to be tested for HIV compared to their urban counterparts [41–43]. These geographic disparities suggest that targeted interventions in underperforming regions could be pivotal in improving overall HIV testing coverage.

Women aged 20–34 years and those with one or more children had higher odds of being ever tested for HIV. This could be attributed to increased healthcare interactions during antenatal and postnatal care, where HIV testing is often promoted. Studies have shown that maternal health services provide critical opportunities for HIV testing and counseling, which likely explains the higher testing rates among women with children [44, 45]. Women with secondary and higher education were more likely to have been tested, reflecting the role of education in enhancing health literacy and awareness. Educated women are more likely to understand the importance of HIV testing and have better access to information about where and how to get tested [46–48].

Compared to those who had never had sex, women who had ever had sex, whether before age 18 (aOR = 3.97, 95% CI = 3.12, 5.05) or at age 18 or older (aOR = 3.78, 95% CI = 2.92, 4.89), had significantly higher odds of ever testing for HIV. This finding reflects the increased likelihood of HIV testing among those at potential risk of sexual transmission; individuals who have never had sex are not at risk of sexually transmitted HIV and are, therefore, less likely to seek testing. Other studies have linked early sexual debut (before age 18) to higher testing rates, often attributing this to women who initiate sexual activity earlier and their greater perceived risk and health-seeking behavior [49–52]. However, this study found that women who initiated sexual activity before or after age 18 had nearly identical odds of testing, suggesting that the act of having sex itself, rather than the timing of sexual debut, is the key determinant of testing behavior.

Women who visited health facilities in the past 12 months had significantly higher odds of being tested, emphasizing the importance of healthcare access in promoting HIV testing [Women who visited health facilities in the past 12 months (aOR = 2.30, 95% CI = 2.12, 2.49) had higher odds of being ever-tested for HIV than those who did not]. Research from other African countries has shown that regular healthcare visits increase the likelihood of HIV testing, which is consistent with this finding [38–40].

On the contextual level, women residing in the Western region had significantly higher odds of being tested than those in the Eastern region. This regional disparity likely reflects differences in healthcare infrastructure, availability of testing services, and public health outreach efforts. Urban regions, such as the Western area, typically have more healthcare facilities and resources dedicated to HIV testing and treatment, which can explain the higher testing rates [37]. Additionally, the higher HIV prevalence in the Western region may contribute to increased testing rates, as greater awareness of HIV risk and targeted public health interventions in areas with higher prevalence can drive testing uptake. Moreover, distance to health facilities emerged as a critical barrier, with women reporting significant issues with access being less likely to be tested. This underscores the need for improving healthcare accessibility in remote areas. The impact of distance on healthcare utilization is well-documented, with numerous studies indicating that proximity to healthcare services significantly influences health-seeking behaviors [38–40]. Future efforts should consider both improving healthcare infrastructure and addressing regional variations in HIV prevalence to ensure equitable access to testing services.

The geographic and socioeconomic disparities in HIV testing observed in this study are consistent with findings from other regions in sub-Saharan Africa. For example, a study conducted in Uganda found that urban residents were more likely to be tested for HIV compared to their rural counterparts, which aligns with our findings of higher testing rates in urban areas like the Western region of Sierra Leone [37]. Moreover, the role of healthcare access as a determinant of HIV testing has been highlighted in several studies. For instance, a study in Nigeria reported that proximity to healthcare facilities and frequent healthcare visits significantly increased the likelihood of HIV testing among women [38–40, 52]. This aligns with our finding that women who visited health facilities in the past 12 months were more likely to be tested.

In contrast, some studies have identified different patterns and determinants of HIV testing. For example, a study in South Africa found that younger women (aged 15–24) were less likely to be tested compared to older age groups, which differs from our finding that women aged 20–34 had higher testing rates [53]. This discrepancy could be due to differences in the implementation of HIV testing programs and the socio-cultural context of the regions studied.

Women who were married or cohabiting exhibited a greater likelihood of having been tested for HIV compared to those who had never been in a relationship, a result that aligns with prior research conducted in sub-Saharan Africa [54, 55]. Marriage and cohabitation frequently foster circumstances that motivate partners to get HIV testing, especially during antenatal care (ANC) appointments, which serve as essential access points for HIV testing services [56]. Women exposed to media were more likely to have had HIV testing, indicating the impact of media initiatives in enhancing awareness of the significance of HIV testing. Prior studies indicate that exposure to HIV-related information via radio, television, or newspapers markedly enhances the probability of testing by mitigating stigma and misconceptions [57]. The utilization of condoms correlated with an increased likelihood of HIV testing, presumably due to condom users being more inclined to pursue health-related actions and recognize their susceptibility to HIV infection, hence motivating them to undergo testing. Moreover, women residing in bigger homes (six or more individuals) exhibited a higher likelihood of having undergone HIV testing compared to those in smaller families. This may be ascribed to the communal characteristics of Sierra Leonean society, wherein larger households potentially possess enhanced access to collective resources and information regarding health services, including HIV testing.

The economic status significantly influenced the likelihood of women being tested for HIV, with those in the middle, richer, and wealthiest categories exhibiting greater odds of having undergone testing compared to their counterparts in the poorest category. This corresponds with findings from earlier research that associate affluent people with improved access to healthcare services and elevated health literacy [58, 59]. Women in the 2013 and 2019 survey years exhibited significantly greater likelihoods of undergoing HIV testing compared to 2008, indicating the influence of national and worldwide initiatives to enhance HIV testing throughout this timeframe. Nonetheless, Muslim women had a lower likelihood of undergoing testing compared to Christian women, a mismatch potentially attributable to cultural and religious variations in health-seeking behaviours. These findings highlight the necessity of overcoming socio-cultural and economic obstacles to HIV testing in Sierra Leone while utilising available resources, such as media and ANC services, to enhance testing rates among all demographic categories.

The findings of this study have several important implications for public health interventions aimed at improving HIV testing strategies among women in Sierra Leone. While increasing testing rates is important, the ultimate goals are to identify people living with HIV, link them to treatment, and reach individuals at high risk of infection to provide prevention services. Given the relatively low levels of ever testing achieved to date (as of 2019), ensuring efficiency in future testing campaigns will be critical by focusing on approaches most likely to reach those at greatest risk of infection. Strategies such as community- and facility-based index testing, which prioritize testing among the contacts of individuals diagnosed with HIV, can be particularly effective. Ensuring high coverage among key populations who are disproportionately affected by HIV is also essential.

Public health initiatives should also prioritize cold spot regions such as Bombali, Koinadugu, and Tonkolili, employing targeted strategies like mobile testing units and community-based testing programs to enhance reach and accessibility. Increasing educational efforts and health literacy programs, particularly in rural and underserved areas, could improve testing uptake. Tailored messages that address the specific barriers and facilitators identified in this study, along with media campaigns and digital platforms, can help disseminate information to broader audiences, especially among younger and more educated women. Improving healthcare infrastructure, reducing barriers to accessing health facilities, and integrating HIV testing with other routine health services remain crucial components of a comprehensive strategy. In general, this study highlights significant geographic variations and determinants of HIV testing among women in Sierra Leone. The findings underscore the importance of targeted interventions, educational efforts, and improved healthcare access to enhance HIV testing rates. By addressing the identified barriers and leveraging the facilitators, public health programs can make substantial progress towards achieving higher HIV testing coverage and ultimately improving HIV prevention and care in Sierra Leone.

### Strengths and limitations

This study’s strengths include comprehensive spatial analysis using spatial autocorrelation and Empirical Bayesian Kriging (EBK), providing detailed insights into the geographic variations in HIV testing rates, and identifying clusters of high and low testing regions. Additionally, the use of mixed-effect multi-level binary logistic regression analysis allowed for a nuanced understanding of both individual and contextual factors associated with HIV testing, effectively separating individual-level effects from community-level influences. The longitudinal aspect of the study, analyzing data from three different Sierra Leone Demographic and Health Surveys (2008, 2013, and 2019), enabled the examination of trends over time and strengthened the ability to identify consistent patterns and changes in HIV testing behavior. The large sample size of 39,606 women provided sufficient power to detect significant associations and variations, enhancing the reliability and generalizability of the findings.

Several limitations should be noted. Ever-tested for HIV is a cumulative indicator, meaning that once an individual is tested, they cannot revert to untested. This limits the ability to measure recent testing behaviors or the frequency of testing, which are more relevant for individuals at ongoing risk of HIV transmission. The cross-sectional design of each dataset limits causal inference between determinants and HIV testing rates, and potential reporting bias may affect accuracy. Some contextual factors, such as cultural beliefs and local healthcare policies, were not fully captured, and the analysis was restricted to variables available in the DHS datasets.

Furthermore, Sierra Leone has a mixed HIV epidemic, and key populations who are disproportionately affected by HIV are not well represented in DHS surveys. Their spatial distribution and uptake of testing may be equally or even more important than general population testing but could not be adequately analyzed in this study. Additionally, the findings may not be directly generalizable to other countries with different socio-cultural contexts, underscoring the need for comparative studies across countries and targeted research on key populations.

## Conclusion

Our findings show a significant increase in HIV testing among women aged 15–49 in Sierra Leone. This study also revealed significant geographic disparities in HIV testing rates among women aged 15–49 in Sierra Leone across the years 2008, 2013, and 2019. Throughout these years, the Western urban area consistently stood out as a hotspot, highlighting key public health intervention zones. Conversely, Bombali, Koinadugu, and Tonkolili districts were frequently identified as cold spots, indicating substantial barriers to accessing HIV testing services in these areas. Factors such as age, education, marital status, media exposure, parity, sexual activity, religion, healthcare utilization, condom use, STI history, household size, household head gender, wealth index, residence, and survey year were associated with HIV testing. These findings suggest that improving healthcare access, establishing HIV testing centers, promoting health literacy, and addressing regional disparities through targeted interventions could significantly boost HIV testing rates among women in Sierra Leone.

## Electronic supplementary material

Below is the link to the electronic supplementary material.

Supplementary Material 1

Supplementary Material 2

Supplementary Material 3

## Data Availability

The data used for this study is freely available at http://dhsprogram.com/data/available-datasets.cfm.

## Abbreviations

aOR: Adjusted Odds Ratio
CI: Confidence Interval
DHS: Demographic and Health Survey
SLDHS: Sierra Leone Demographic and Health Survey
HIV: Human Immuno Virus
MEASURE DHS: Monitoring and Evaluation to Assess and Use Results Demographic and Health Surveys
STROBE: Strengthening the Reporting of Observational Studies in Epidemiology
