# Supplementary material for: Geographic variations and determinants of ever-tested for HIV among women aged 15–49 in Sierra Leone: a spatial and multi-level analysis
Source: BMC Public Health. 2025 Mar 11;25:961. doi: 10.1186/s12889-025-22079-7 (PMC11895344; doi:10.1186/s12889-025-22079-7)
Supplement: Supplementary file 1 — Supplementary Material 1 [file 12889_2025_22079_MOESM1_ESM.docx]

**Supplementary File- 1: Statistical Analysis**

**Contents**

**S1.0. Spatial Analysis of HIV testing uptake in women aged 15-49………………1**

**S1.1. Multi-level binary logistic regression………………………………………..2**

**S1.2 References**

**S1.0 Spatial Analysis of HIV testing uptake in women aged 15-49**

First, the Spatial autocorrelation (Global Moran's I) statistic measure was used to evaluate whether the HIV testing patterns were dispersed, clustered, or randomly distributed from 2008 to 2019. Moran's I is a spatial statistic used to measure spatial autocorrelation by taking the entire data set and producing a single output value that ranges from − 1 to + 1(1). A positive value for Moran's Index indicates a clustered pattern of HIV testing, while a negative value for Moran's Index indicates a dispersed pattern, and HIV testing is distributed randomly if the Index value is zero. Next, Getis-Ord GI* statistics were used to investigate the spatial autocorrelation patterns within the study areas for all three survey years. The realization of this result was made possible by computing the GI* statistic for each location. A p-value greater than 0.05 is used to establish a cluster of statistical significance. Areas with a low Geographic Information (GI) value are associated with "cold spots," which imply a lower chance of HIV testing, while areas with a high GI* value are indicative of "hotspots"—defined by an enhanced likelihood of HIV testing (1).

Finally, the Empirical Bayesian Kriging (EBK) technique was used to create interpolation maps to predict the magnitude of HIV testing for 2008, 2013, and 2019. The magnitude of the HIV testing distribution was determined using the Ordinary Kriging method of Interpolation. This technique predicts values in regions where no data points have been sampled based on how far those locations are from sentinel locations where measurements have been taken (2, 3). The technique uses spatial autocorrelation to identify spatial patterns in the data and predict underlying dependency patterns(3, 4).

**S1.1 Multi-level binary logistic regression**

We computed the Variance Inflation Factor (VIF) to assess multicollinearity among the independent variables. All VIF values were below **2**, indicating minimal multicollinearity. Therefore, we can conclude that multicollinearity is not a concern, and we can confidently interpret the regression coefficients.

Thus, we used a four-modeled approach for the regression analysis, using only variables with a p-value less than or equal to 0.05. The first model (Model I) had no explanatory variables, and its results demonstrated the variations in HIV testing attributed to the clustering at the primary sampling units (PSU). The individual level variables were placed in Model II, whereas Model III contained the contextual level variables. Model IV contained all the explanatory variables.

The mixed-effect regression analysis had fixed-effect and random effects as its results. The fixed-effect results showed the association between the explanatory variables and HIV testing. We presented the results as an adjusted odds ratio (aOR) with their respective 95% CI. On the other hand, the random effect results imply variations in HIV testing. This variation was indicated by the intra-cluster correlation coefficient (ICC) values across the four models. We used the lowest value of the Akaike Information Criterion (AIC) and the highest log likelihood value to select the best-fitted model for the study. Therefore, the last model, Model IV, was adjudged as the best model from which the fixed effect results were described and discussed. Before generating the results, we weighted the dataset, and the surveyset command in Stata was used throughout the study.

However, we acknowledge the growing interest in modeling prevalence ratios directly, particularly in cross-sectional studies where the outcome of interest is relatively common (i.e., prevalence >10%), as is the case in our study. While logistic regression provides odds ratios, which may overestimate the relative risk when the outcome is common, alternative approaches such as log-binomial regression or Poisson regression with robust variance estimation are often used to estimate prevalence ratios. However, these methods can be challenging to implement when incorporating spatial components or hierarchical structures, as they may not easily accommodate random effects or spatial dependencies. To address this limitation, future analyses could explore generalized linear mixed models (GLMMs) with a log link function to directly estimate prevalence ratios while accounting for the multilevel structure of the data. Additionally, spatial regression techniques, such as Bayesian spatial models, could potentially be adapted to estimate prevalence ratios in settings with spatial components. While these methods were not used in the current analysis, we recognize their potential and encourage further research in this area.

**S1.2. References**

1. Tsai P-J, Lin M-L, Chu C-M, Perng C-H. Spatial autocorrelation analysis of health care hotspots in Taiwan in 2006. BMC Public Health. 2009;9:1-13.

2. Gelibo T, Lulseged S, Eshetu F, Abdella S, Melaku Z, Ajiboye S, et al. Spatial distribution and determinants of HIV prevalence among adults in urban Ethiopia: Findings from the Ethiopia Population-based HIV Impact Assessment Survey (2017–2018). Plos one. 2022;17(7):e0271221.

3. Shimbre MS, Tunja A, Bodicha BB, Belete AG, Hailgebereal S, Fornah L, et al. Spatial mapping and predictors of ever-tested for HIV in adolescent girls and young women in Ethiopia. Frontiers in Public Health. 2024;12:1337354.

4. Krivoruchko K. Empirical bayesian kriging. ArcUser Fall. 2012;6(10):1145.
