# Supplementary material for: Geographic variations and determinants of ever-tested for HIV among women aged 15–49 in Sierra Leone: a spatial and multi-level analysis
Source: BMC Public Health. 2025 Mar 11;25:961. doi: 10.1186/s12889-025-22079-7 (PMC11895344; doi:10.1186/s12889-025-22079-7)
Supplement: Supplementary file 2 — Supplementary Material 2 [file 12889_2025_22079_MOESM2_ESM.docx]

**Supplementary File 2: Background Characteristics and Bi-variate Analysis**

**Table of Contents**

**S2.0 Background characteristics of women in Sierra Leone using pooled datasets 2008,2013 and 2019……………………………………………………………………………………….2**

**S2.1 Bivariate results of the association between the explanatory variables and ever-tested HIV…………………………………………………………………………………………….5**

**Tables:**

**Table S2.0: Background characteristics of women in Sierra Leone using pooled datasets(2008,2013 and 2019)……………………………………………………………..2-5**

**Table S2.1: Bivariable analysis of HIV testing among women in Sierra Leone…………..6-9**

**S2.0 Background characteristics of women in Sierra Leone using pooled datasets 2008,2013 and 2019**

**Table S2.0: Background characteristics of women in Sierra Leone using pooled datasets 2008,2013 and 2019 (n=39, 606)**

| **Variables** | **Weighted sample** | **Weighted percentage** |
| --- | --- | --- |
| **Women's age (years)** |  |  |
| 15-19 | 8,503 | 21.47 |
| 20-24 | 6,498 | 16.41 |
| 25-29 | 7,214 | 18.22 |
| 30-34 | 5,273 | 13.31 |
| 35-39 | 5,615 | 14.18 |
| 40-44 | 3,351 | 8.46 |
| 45-49 | 3,152 | 7.96 |
| **Educational attainment** |  |  |
| No education | 21,235 | 53.62 |
| Primary | 5,393 | 13.62 |
| Secondary | 11,629 | 29.36 |
| Higher | 1,348 | 3.40 |
| **Current working status** |  |  |
| Not working | 12,226 | 30.87 |
| Working | 27,380 | 69.13 |
| **Marital status** |  |  |
| Never in union | 11,187 | 28.24 |
| Married/cohabiting | 26,143 | 66.01 |
| Previously married | 2,276 | 5.75 |
| **Exposed to media** |  |  |
| No | 38,579 | 97.41 |
| Yes | 1,027 | 2.59 |
| **Covered by Health insurance** |  |  |
| No | 38,707 | 97.73 |
| Yes | 899 | 2.27 |
| **Parity** |  |  |
| Zero | 9,710 | 24.52 |
| One | 5,951 | 15.03 |
| Two or more | 23,945 | 60.46 |
| **Age at first sex** |  |  |
| Not had sex | 3,528 | 8.91 |
| Below 18 years | 23,506 | 59.35 |
| 18+ | 12,573 | 31.74 |
| **Religion** |  |  |
| Christians | 8,828 | 22.29 |
| Muslims | 30,650 | 77.39 |
| Others | 128 | 0.32 |
| **Visited health facility last 12 months** |  |  |
| No | 20,539 | 51.86 |
| Yes | 19,067 | 48.14 |
| **Multiple sexual partners** |  |  |
| No | 37,789 | 95.41 |
| Yes | 1,817 | 4.59 |
| **Condom use** |  |  |
| No | 38,999 | 98.47 |
| Yes | 607 | 1.53 |
| **Had STI** |  |  |
| No | 35,762 | 90.29 |
| Yes | 3,844 | 9.71 |
| **Genital discharge** |  |  |
| No | 33,503 | 84.59 |
| Yes | 6,103 | 15.41 |
| **Genital sore** |  |  |
| No | 35,401 | 89.38 |
| Yes | 4,205 | 10.62 |
| **Distance to a health facility** |  |  |
| No problem | 6,678 | 16.86 |
| Big problem | 32,928 | 83.14 |
| **Household size** |  |  |
| Five and below | 14,280 | 36.06 |
| Six or more | 25,326 | 63.94 |
| **Sex of household head** |  |  |
| Male | 28,419 | 71.75 |
| Female | 11,187 | 28.25 |
| **Wealth index** |  |  |
| Poorest | 7,210 | 18.20 |
| Poorer | 7,246 | 18.29 |
| Middle | 7,522 | 18.99 |
| Richer | 8,245 | 20.82 |
| Richest | 9,384 | 23.69 |
| **Place of residence** |  |  |
| Urban | 15,751 | 39.77 |
| Rural | 23,855 | 60.23 |
| **Region** |  |  |
| Eastern | 8,008 | 20.22 |
| Northern | 12,610 | 31.84 |
| Northwestern | 7,564 | 19.10 |
| Southern | 7,645 | 19.30 |
| Western | 3,780 | 9.54 |

**S2.1 Bivariate results of the association between the explanatory variables and ever-tested HIV**

Being ever tested for HIV was high among women aged 25-29 (58.2%), women who had higher education 74.4%, women who were working 47.6%, women who were married/ cohabiting 50.7%, women who were exposed to media 59.1%, women covered by health insurance 63,4%, women with one child 61.9%, women who had sex below 18 (49.9%), women who visited a health facility in the last 12 months 59.3%, women with multiple sexual partners 52.8%, women who were in the richest wealth index 53.4%, women who lived in urban areas 51.9% and women who live in the Western region 65.3%. All the explanatory variables were significantly associated with being ever-tested for HIV among women aged 15-49 years in Sierra Leone at p<0.05.

**Table S2.1: Bivariable analysis of HIV testing among women in Sierra Leone**

| **Variables** | **Ever-tested HIV** | **p-value** |
| --- | --- | --- |
| **Pooled Prevalence** | 45.5% [44.2,46.8] |  |
| **Women's age (years)** |  | <0.001 |
| 15-19 | 22.8 [21.5,24.2] |  |
| 20-24 | 54.2 [52.3,56.1] |  |
| 25-29 | 58.2 [56.0,60.3] |  |
| 30-34 | 57.9 [55.8,60.0] |  |
| 35-39 | 50.7 [48.5,52.8] |  |
| 40-44 | 42.8 [40.6,45.1] |  |
| 45-49 | 32.7 [30.4,35.0] |  |
| **Educational attainment** |  | <0.001 |
| No education | 42.7 [41.0,44.4] |  |
| Primary | 44.9 [42.6,47.3] |  |
| Secondary | 47.6 [46.2,49.0] |  |
| Higher | 74.4 [71.2,77.3] |  |
| **Current working status** |  | <0.001 |
| Not working | 40.8 [39.3,42.2] |  |
| Working | 47.6 [46.1,49.2] |  |
| **Marital status** |  | <0.001 |
| Never in union | 32.8 [31.3,34.4] |  |
| Married/cohabiting | 50.7 [49.1,52.4] |  |
| Previously married | 47.7 [44.9,50.5] |  |
| **Media exposure** |  | <0.001 |
| No | 45.1 [43.9,46.4] |  |
| Yes | 59.1 [54.9,63.1] |  |
| **Covered by Health insurance** |  | <0.001 |
| No | 45.1 [43.8,46.4] |  |
| Yes | 63.4 [57.7,68.7] |  |
| **Parity** |  | <0.001 |
| Zero | 19.4 [18.2,20.7] |  |
| One | 61.9 [59.9,63.8] |  |
| Two or more | 52.0 [50.4,53.6] |  |
| **Age at first sex** |  | <0.001 |
| Not had sex | 6.7 [5.5,8.2] |  |
| Below 18 years | 49.9 [48.4,51.4] |  |
| 18+ | 48.2 [46.3,50.0]] |  |
| **Religion** |  | <0.001 |
| Christians | 50.0 [48.0,52.0] |  |
| Muslims | 44.2 [42.8,45.7] |  |
| Others | 41.3 [31.1,52.3] |  |
| **Visited health facility last 12 months** |  | <0.001 |
| No | 32.7 [31.4,34.1] |  |
| Yes | 59.3 [57.6,60.9] |  |
| **Multiple sexual partners** |  | <0.001 |
| No | 45.1 [43.9,46.4] |  |
| Yes | 52.8 [49.7,55.9] |  |
| **Condom use** |  | <0.001 |
| No | 45.3 [44.1,46.6] |  |
| Yes | 56.1 [51.0,61.1] |  |
| **Had STI** |  | <0.001 |
| No | 44.2 [42.9,45.4] |  |
| Yes | 58.0 [55.4,60.6] |  |
| **Genital discharge** |  | <0.001 |
| No | 43.9 [42.6,45.2] |  |
| Yes | 54.1 [52.0,56.2] |  |
| **Genital sore** |  | <0.001 |
| No | 44.8 [43.5,46.0] |  |
| Yes | 51.6 [49.0,54.2] |  |
| **Distance to a health facility** |  | <0.001 |
| No problem | 54.1 [52.2,56.0] |  |
| Big problem | 43.8 [42.3,45.2] |  |
| **Household size** |  | <0.001 |
| Five and below | 48.0 [46.5,49.5] |  |
| Six or more | 44.1 [42.7,45.5] |  |
| **Sex of household head** |  | 0.002 |
| Male | 44.7 [43.3,46.1] |  |
| Female | 47.6 [46.1,49.1] |  |
| **Wealth index** |  | <0.001 |
| Poorest | 40.6 [38.3,43.0] |  |
| Poorer | 41.4 [39.1,43.8] |  |
| Middle | 42.2 [40.0,44.5] |  |
| Richer | 47.4 [45.5,49.3] |  |
| Richest | 53.4 [51.7,55.0] |  |
| **Place of residence** |  | <0.001 |
| Urban | 51.9 [50.2,53.5] |  |
| Rural | 41.3 [39.5,43.1] |  |
| **Region** |  | <0.001 |
| Eastern | 45.7 [43.0,48.4] |  |
| Northern | 37.9 [35.0,40.8] |  |
| Northwestern | 44.0 [41.7,46.3] |  |
| Southern | 49.5 [47.6,51.5] |  |
| Western | 65.3 [62.5,68.1] |  |
