# Supplementary material for: Geographic variations and determinants of ever-tested for HIV among women aged 15–49 in Sierra Leone: a spatial and multi-level analysis
Source: BMC Public Health. 2025 Mar 11;25:961. doi: 10.1186/s12889-025-22079-7 (PMC11895344; doi:10.1186/s12889-025-22079-7)
Supplement: Supplementary file 3 — Supplementary Material 3 [file 12889_2025_22079_MOESM3_ESM.docx]

**Supplementary File 3: Spatial Pattern of Ever-tested for HIV among women aged 15-49**

**Table of Content**

**Table:**

**Table S1. Spatial autocorrelation of HIV testing among women aged 15-49 in Sierra Leone, 2008,2013 and 2019………………………………………………………………………….1**

**Table S1. Spatial autocorrelation of HIV testing among women aged 15-49 in Sierra Leone, 2008,2013 and 2019**

| **Survey** | **Moran's Index** | **Expected Moran's Index** | **Z-score** | **p-value** |
| --- | --- | --- | --- | --- |
| **SLDHS 2008** | 1.6204 | -0.0028 | 3.7612 | 0.0002 |
| **SLDHS 2013** | 0.2318 | -0.0023 | 6.7781 | <0.0001 |
| **SLDSH 2019** | 0.3142 | -0.0018 | 9.9184 | <0.0001 |

**SLDHS:** Sierra Leone Demographic and Health Survey; HIV: Human Immunodeficiency Virus
